# Transvenous embolization using the Amplatzer Vascular Plug II in patent ductus arteriosus concomitant with Stanford type B aortic dissection: A case report

**DOI:** 10.1097/MD.0000000000033936

**Published:** 2023-07-07

**Authors:** Jong Hun Woo, Jongjoon Shim, Jae Myeong Lee

**Affiliations:** a Department of Radiology, University of Soonchunhyang College of Medicine, Soonchunhyang University Bucheon Hospital, Bucheon-si, Gyeonggi-do, Republic of Korea.

**Keywords:** Amplatzer Vascular Plug II, aortic dissection, patent ductus arteriosus, transvenous embolization

## Abstract

Thoracic endovascular aneurysm repair (TEVAR) is commonly used to treat Stanford type B aortic dissections. However, coexistence of aortic dissection and patent ductus arteriosus (PDA) is an extremely rare phenomenon, and TEVAR alone is insufficient for treatment. Herein, a case of endovascular treatment in a patient with both aortic dissection and PDA is reported.

**Patient concerns:**

A 31-year-old woman presented to the authors’ hospital with chest pain extending to the back. At presentation, her blood pressure was 130/70 mm Hg. Her father, brother, and uncle were all diagnosed with aortic dissection.

**Diagnoses:**

Computed tomography (CT) revealed Stanford type B aortic dissection from the aortic arch to the infrarenal abdominal aorta; however, PDA was incidentally identified.

**Interventions:**

TEVAR was immediately performed. Follow-up CT scan performed 2 months later did not reveal any thrombosis or remodeling of the false lumen, and the PDA remained open. Therefore, an additional PDA embolization procedure was performed using the Amplatzer Vascular Plug II via the transvenous route.

**Outcomes:**

On follow-up CT performed 6 months after PDA embolization, successful remodeling, and shrinkage of the false lumen were observed, and PDA closure was confirmed.

**Lessons:**

If Stanford type B aortic dissection and PDA coexist, TEVAR alone may not be a sufficient treatment and additional PDA embolization may be required. In the present case, transvenous embolization of PDA using an Amplatzer Vascular Plug II was safe and effective.

## 1. Introduction

Ductus arteriosus is a fetal vascular structure that connects the trunk of the pulmonary artery to the proximal segment of the descending thoracic aorta. It closes naturally within 24 to 48 hours after birth. Patent ductus arteriosus (PDA) is characterized by persistent opening of the ductus arteriosus. PDA accounts for 5 to 10% of all congenital heart diseases and is considered to be among the most frequently occurring.^[1–4]^

PDAs provide continuous left-to-right shunting. Therefore, the presence of PDA can cause heart failure and dyspnea due to pulmonary hypertension, and a large PDA can even cause Eisenmenger syndrome. Thus, treatment is necessary in cases of symptomatic PDA.^[3,5]^

Coexistence of PDA and aortic dissection is very rare, with only a few case reports describing the phenomenon.^[6–8]^ For Stanford type B aortic dissection, thoracic endovascular aortic repair (TEVAR) is the preferred treatment option over open surgery because of its minimally invasive nature, lower mortality rates, and fewer complications. The treatment principle of TEVAR is to induce thrombus formation and remodel the false lumen by deploying a stent graft to seal the entry tear site.^[9,10]^

To the best of our knowledge, there have been no reported cases in which PDA hindered false lumen thrombosis and remodeling after TEVAR was performed for the treatment of aortic dissection. We performed TEVAR in a patient with coexisting Stanford type B aortic dissection and PDA, and then additionally performed PDA embolization to induce thrombosis and remodeling of the false lumen. In general, transcatheter embolization of PDA is performed using the Amplatzer Duct Occluder device as the transarterial route.^[11]^ However, we performed transvenous PDA embolization using the Amplatzer Vascular Plug II, which is described in the present report.

## 2. Case report

A 31-year-old woman presented to the emergency room with sudden chest pain, which she experienced in the morning, extending from the chest to the back. The patient was otherwise healthy and had no relevant medical history; her blood pressure was initially measured to be 130/70 mm Hg. However, she had an unusual family history; more specifically, her father, brother, and uncle were all diagnosed with aortic dissection.

**Figure 1.**
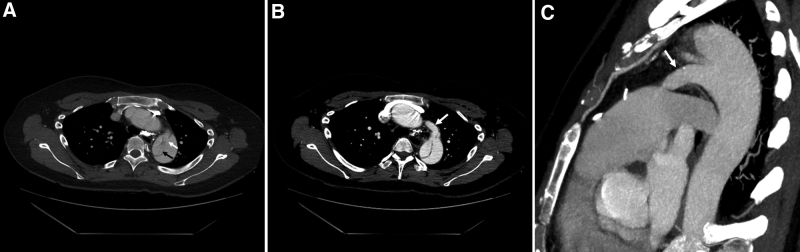
(A) CT revealing the dissection flap (black arrow) and entry tear site (white arrow) of the descending thoracic aorta. (B) Axial CT revealing PDA (arrow connected to the proximal descending thoracic aorta. (C) Sagittal CT revealing PDA (arrow), which has a narrow connection to the pulmonary trunk and a wide connection to the aorta. CT = computed tomography, PDA = patent ductus arteriosus.

Chest radiography revealed mediastinal widening. Considering her symptoms, family history, and chest radiographic findings, aortic dissection was suspected. Enhanced computed tomography (CT) revealed aortic dissection from the descending thoracic aorta to the infrarenal abdominal aorta. The entry tear site was located distal to the left subclavian artery os (Fig. 1A).

However, a PDA was identified in addition to aortic dissection (Fig. 1B). The PDA was elongated with a wide connection to the thoracic aorta and a very narrow connection to the pulmonary trunk (Fig. 1C). The diameter of the PDA was approximately10 mm.

Despite being a Stanford type B aortic dissection, it was decided to perform TEVAR due to the extensive scope of the dissection. It was believed that relying solely on medical treatment would have some limitations. It was also believed that the coexisting PDA could be sealed using TEVAR.

TEVAR was performed immediately. The right common femoral artery was punctured under ultrasonographic guidance. A 20-F introducer sheath was inserted and a 32 mm × 150 mm stent graft (S&G Biotech, Seongnam, Korea) was introduced through introducer sheath. A stent graft was installed from the distal portion of the left subclavian artery os to the proximal portion of the descending thoracic aorta, and flow through the PDA was not observed on subsequent aortography.

**Figure 2.**
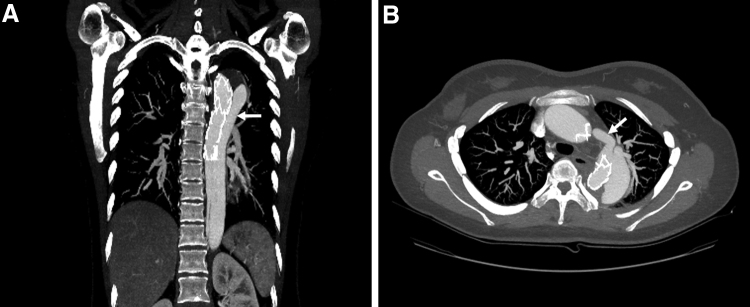
(A) Coronal CT performed 2 months after TEVAR showing a false lumen (arrow) filled with contrast agent, without inducing thrombosis. (B) Axial CT showing that the PDA (arrow) remains open. CT = computed tomography, PDA = patent ductus arteriosus, TEVAR = thoracic endovascular aortic repair.

However, on follow-up CT performed 2 months later, thrombosis and remodeling of the false lumen were not induced (Fig. 2A), and the PDA remained open in connection with the false lumen (Fig. 2B). It was then decided to perform PDA embolization because the presence of the PDA interfered with thrombosis and remodeling of the false lumen.

**Figure 3.**
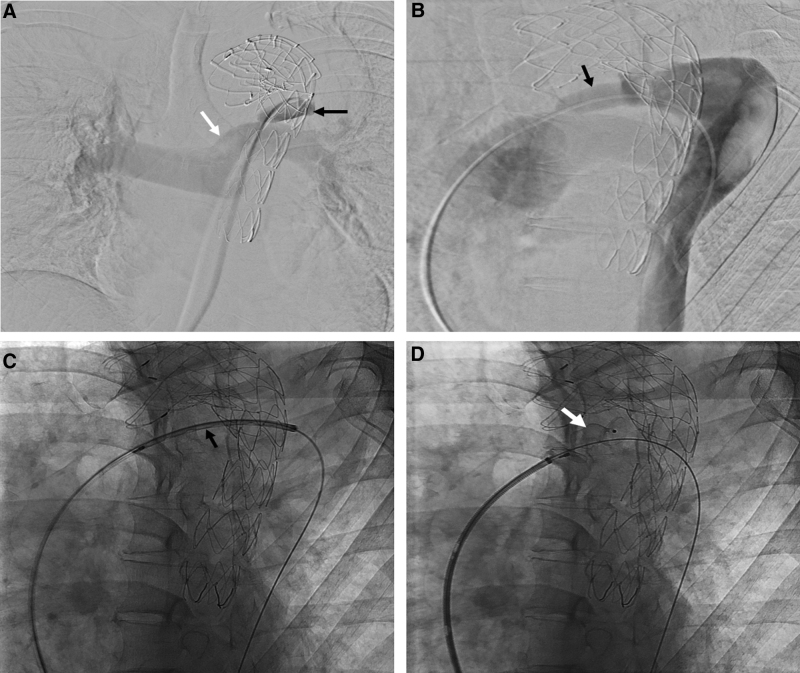
(A) Angiography performed using an angiographic catheter placed inside the PDA revealing the pulmonary trunk (white arrow) connected to the PDA (black arrow). (B) Angiography performed using an angiographic catheter placed in the false lumen of the descending thoracic aorta showing contrast medium flowing from the false lumen, through the PDA (arrow), into the pulmonary trunk. (C) Fluoroscopy showing a 7-F guiding sheath in the PDA and a vascular plug (arrow) being delivered within the guiding sheath. (D) Fluoroscopy showing the Amplatzer Vascular Plug II (arrow) well installed in the PDA. PDA = patent ductus arteriosus.

PDA embolization was performed 3 months after TEVAR. The right common femoral vein was punctured under ultrasonographic guidance. A 7-F guiding sheath (Flexor Shuttle Guiding Sheath, Cook Medical, Bloomington, IN) was then inserted into the pulmonary trunk through the right common femoral vein. Catheterization of the PDA was performed using a 5 -F angiographic catheter (Davis Angiographic Catheter, Cook Medical) and 0.035-in hydrophilic guide wire (Radifocus, Terumo, Tokyo, Japan) (Fig. 3A). Angiography was performed after introducing an angiographic catheter into the false lumen of the aorta. Angiography revealed that contrast agent flowed from the false lumen of the aorta to the pulmonary trunk through the PDA (Fig. 3B). The guide wire was replaced with a stiff type (Radifocus, Terumo, Tokyo, Japan), and a 7-F guiding sheath was introduced into the PDA along the guide wire and angiographic catheter located within the false lumen of the aorta (Fig. 3C). Subsequently, a 14 mm vascular plug (Amplatzer Vascular Plug II, Abbott Medical, Plymouth, MN) was introduced through the sheath, and a vascular plug was placed in the PDA (Fig. 3D).

**Figure 4.**
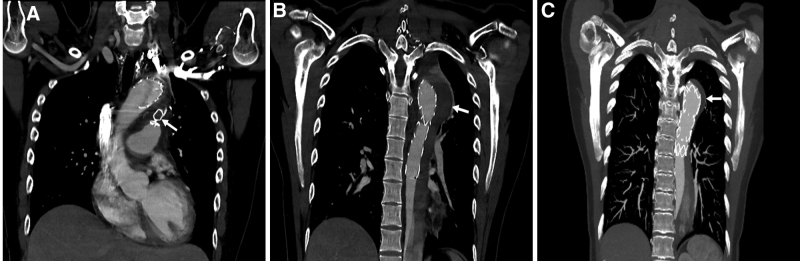
(A and B) Coronal CT performed 3 days after PDA embolization showing a normally positioned vascular plug (arrow in A) in PDA and a false lumen filled with a thrombus (arrow in B) within the PDA. (C) Coronal CT performed 6 months after PDA embolization showing a successfully remodeled and shrunken false lumen (arrow). CT = computed tomography, PDA = patent ductus arteriosus.

On CT performed 3 days after PDA embolization, the vascular plug was normally located inside the PDA (Fig. 4A) and the false lumen of the descending thoracic aorta was completely thrombosed (Fig. 4B). The patient was discharged on the following day.

Six months after PDA embolization, the patient underwent follow-up CT, which revealed that the false lumen was markedly shrunken and blood flow through the PDA was not evident (Fig. 4C).

## 3. Discussion

The patient was unaware of the presence of PDA until she presented to our hospital’s emergency department and underwent CT. PDA was incidentally discovered during confirmation of aortic dissection. The patient did not experience symptoms, such as dyspnea, due to pulmonary hypertension or heart failure. No evidence of cardiac enlargement or pulmonary congestion was observed on CT imaging. Additionally, cardiac echocardiography performed during hospitalization revealed normal cardiac function. Despite a maximum PDA diameter of approximately 10 mm, it was suggested that there was minimal left-to-right shunting due to the narrow connection to the pulmonary trunk.

Porstmann et al performed the first transcatheter closure of PDA in 1967, which has remained the preferred treatment of choice to date, owing to its advantages of minor trauma and shorter recovery time.^[12,13]^

Embolic devices used for PDA embolization are diverse. For small PDAs, embolization can be performed using coils.^[14]^ However, in our case, the connection between the PDA and aorta was wide; therefore, the risk of coils migrating to the false lumen of the aorta was high. For moderate to large PDAs, the Amplatzer Duct Occluder is often used.^[11,15]^ However, in our patient’s case, the shape of the PDA was elongated; as such, we believed the Amplatzer Vascular Plug II was more suitable. To install the Amplatzer Vascular Plug II, a 6- or 7-F guiding sheath must be inserted into the PDA. In our patient, the PDA was directed upward from the roof of the pulmonary trunk. Therefore, the transvenous approach was deemed an easier option and selected for installation of the vascular plug. The insertion of the guiding sheath into the PDA through the aorta was deemed challenging because of a curved pathway, which could have hindered the insertion of the guiding sheath and delivery of the vascular plug.

As mentioned, coexistence of aortic dissection and PDA is a very rare phenomenon. In most cases, false lumen thrombosis induction and PDA closure were achieved by performing TEVAR only.^[8,16]^ However, our case demonstrated that, in situations in which PDA and aortic dissection coexist, TEVAR alone may not be sufficient for thrombosis and remodeling of the false lumen or PDA closure. TEVAR induces remodeling by causing stasis of blood flow within the false lumen by sealing the entry tear site.^[9,10]^ However, in the presence of PDA, blood flow stasis in the false lumen of the aorta may not be induced, and thrombosis or remodeling in the false lumen may eventually be hindered. Therefore, PDA embolization should be considered in addition to TEVAR when PDA coexists with Stanford type B aortic dissection.

In conclusion, closure of a PDA using the Amplatzer Vascular Plug II via the transvenous route was safe and feasible and effectively achieved complete occlusion. As in the case described herein, when PDA and Stanford type B aortic dissection coexist, implementing PDA embolization in addition to TEVAR can increase treatment success.

## Author contributions

**Conceptualization:** Jong Hun Woo, Jongjoon Shim, Jae Myeong Lee.

**Formal analysis:** Jongjoon Shim, Jae Myeong Lee.

**Funding acquisition:** Jong Hun Woo, Jongjoon Shim.

**Investigation:** Jongjoon Shim.

**Resources:** Jong Hun Woo, Jae Myeong Lee.

**Supervision:** Jongjoon Shim, Jae Myeong Lee.

**Writing – original draft**: Jong Hun Woo.

**Writing – review & editing:** Jongjoon Shim, Jae Myeong Lee.
